# Why Would You Choose to Do an Extreme Sport?

**DOI:** 10.3390/jfmk6030065

**Published:** 2021-07-26

**Authors:** Giuseppe Musumeci

**Affiliations:** 1 Department of Biomedical and Biotechnological Sciences, Human, Histology and Movement Science Section, University of Catania, Via S. Sofia n°87, 95123 Catania, Italy; graziamaugeri@unict.it or g.musumeci@unict.it; Tel.: +39-095-378-2043; 2 Research Center on Motor Activities (CRAM), University of Catania, Via S. Sofia n°97, 95123 Catania, Italy; 3 Department of Biology, Sbarro Institute for Cancer Research and Molecular Medicine, College of Science and Technology, Temple University, Philadelphia, PA 19122, USA

Why do so many athletes keep practicing extreme sports, even though they know the danger of risking their lives? Why is our body addicted to these strong emotions? I will try to address these questions in this short editorial. 

The thrills given by extreme sports attract many individuals seeking excitement. Many of these extreme sports like snowboarding, surfing, skateboarding, rock climbing, bungee jumping, skydiving, and others, allow one to feel the freedom to challenge yourself, both physically and psychologically, and to perform any type of freestyling that would be nauseating to athletes. However, almost all extreme sports have some elements that could endanger an athlete’s life in comparison to traditional sports. These sports could be defined as “extreme” due to their tendency to be dangerous if not performed carefully or with the right equipment [1]. After all, to experience the true “adrenaline kick,” these sports must be dangerous. Serious injuries are common among adrenaline junkies and many fatalities are reported every year. To give an example of this phenomenon according to the report of the United States Parachute Association, more than twenty people a year die due to parachuting alone. The effort required by these sports is great, but the supply of adrenaline and other hormones is sufficient to avoid tiredness resulting from exercise. The adrenaline rush increases the acceleration of blood flows to the muscles and brain, relaxes the muscles, and lastly helps with the conversion of glycogen into glucose in the liver. For every extreme sports athlete, this adrenaline rush is never enough since they are always seeking stronger emotions.

This kind of feeling cannot be otherwise experienced and many of these extreme sports athletes do not even consider a life without the excitement of these powerful moments. Furthermore, extreme sports have the capacity to establish a strong bond between individuals, thanks to the dangerous elements of the activity that requires a high level of trust between people. Consequently, this kind of friendship bond has a good impact on mental health [2].

The typical challenges and performances of the so-called “extreme sports” draw the attention of the spectators, growing the interest of researchers in this kind of behavior. The reasons why risk-lovers are attracted to challenges in dangerous places, or to the possibility of facing the unknown or even to the extreme conditions in which it must be lived, are strictly related to their interpretation of life, to their need of challenging life and to have complete control of the most uncertain situations [3].

These aspects need to be monitored and reworked in case of predominance of self-destructive tendencies, or when evaluating self-capacities. In this situation, the tendency to underestimate the risk could hide the overestimation of the self, or a devaluation of life caused by a non-depressive mental state that can lead to a latently desired death [1]. However, most extreme sports enthusiasts are not driven by self-destructive tendencies. One of the most important aspects of extreme sports that fascinates people is the possibility to live experiences that make you feel alive in a way out of the ordinary, that generate euphory described with expressions like “feeling in the eye of the storm” or “look I’m getting” or “feel the adrenaline rush”.

Some studies tried to explain the neuropsychological reasons that may lead some people more than others to look for “no limits” experiences. These studies found a correlation between the ability of certain activities to enhance adrenaline’s secretion, the need to take risks, and the inclination to seek extreme experiences. This chemical response is closely related to the so-called “fight or flight”, which is able to generate chills reported as “pleasant” in those who frequently seek these kinds of experience. The feeling of imminent danger elicited by these extreme sports activates the survival mechanisms in response to stress in order to face the event through neurophysiological changes broadly acknowledged by the literature [2].

However, it is possible to activate the “fight or flight” response in the average population even with activities that guarantee great safety and that allow people to deal with uncertainties or changes with respect to the usual point of reference: like the small challenges to daily habits of some game at the funfair that are able to elicit a pleasant, and safe, euphory. Emotional experiences on daily life have also been related to the release of neuromediators, which is physiologically activated in several situations faced by the individuals.

In these scenarios, the organism produces a large amount of dopamine which is known to elicit the sensation of pleasure similar to those experienced with alcohol, drugs, or sexual intercourse. Therefore, this explains (along with the presence of adrenaline) the frequent propensity to uncontrollably smile or scream while living those experiences. The common attraction towards these situations has also been studied in relation to a gene mutation that could cause a lower presence of dopamine receptors. This mutation has been found in many people who express attraction to extreme sports; therefore, it was considered among the possible physiological reasons that can explain the tendency to experiment with extreme activities, since the latter would be able to induce the overproduction of dopamine in order to obtain those physiological effects which are physiologically achieved at a lower level of stimulation in people with, otherwise, a greater number of dopaminergic receptors [4].

Many other studies on the typical personality of extreme sports enthusiasts spotted in these people the propensity to seek strong emotions, and this has led to the definition of “sensation seekers”, a psychological aspect very common between paratroopers, free climbers, and other athletes practicing extreme sports or showing addiction to exercise [5]. In a similar context, it is possible to place the psychological studies that have compared the differences between common people and “sensation seekers”. Sensation seekers are characterized by a need to try the extreme, in search of thrills, even though it implies doing dangerous sports.

These kinds of people avoid trivial experiences because they need high-emotional situations (like drug addicts), developing a sort of “shivering tolerance”, forcing them to seek higher doses of emotion every time to reach the same sensation as before. When this occurs, they get used to the same extreme challenge and start looking for a more intense one, to feel the thrill again, risking death just as might happen in drug addiction. In these situations, the need to seek the thrill is combined with a system of values or criminal behaviour tendencies, fuelled by an altered evaluation of life: the result is the pursuit of one’s passion, putting in danger himself and other lives [2].

There are various reasons why it would be interesting to tackle the challenge of extreme sports, but before venturing into them, it is necessary to consider and reflect on the above-discussed arguments. Furthermore, people who want to undertake these sports should be careful about their own and others’ physical integrity, because sport should simply improve the psychophysical abilities of the person and not the other way around.
